# Interprofessional collaboration in palliative dementia care through the eyes of informal caregivers

**DOI:** 10.1177/14713012221098259

**Published:** 2022-05-10

**Authors:** Chandni Khemai, Judith M Meijers, Irma Mujezinovic, Sascha R Bolt, Sabine Pieters, Albine Moser, Jos M G A Schols, Daisy J A Janssen

**Affiliations:** Department of Health Services Research, CAPHRI School for Public Health and Primary Care, Faculty of Health Medicine and Life Sciences, 5211 Maastricht University, Maastricht, Limburg, Netherlands; Department of Health Services Research, CAPHRI School for Public Health and Primary Care, Faculty of Health Medicine and Life Sciences, 5211 Maastricht University, Maastricht, Limburg, Netherlands; Zuyderland Care, 159205 Zuyderland Medical Center, Sittard-Geleen, Limburg, Netherlands; Department of Health Services Research, CAPHRI School for Public Health and Primary Care, Faculty of Health Medicine and Life Sciences, 5211 Maastricht University, Maastricht, Limburg, Netherlands; 5216 Zuyd University of Applied Sciences, Heerlen, Limburg, Netherlands; Department Family Medicine, 5211 Maastricht University, Maastricht, Limburg, Netherlands; Department of Health Services Research, CAPHRI School for Public Health and Primary Care, Faculty of Health Medicine and Life Sciences, 5211 Maastricht University, Maastricht, Limburg, Netherlands; Department of Health Services Research, CAPHRI School for Public Health and Primary Care, Faculty of Health Medicine and Life Sciences, 5211 Maastricht University, Maastricht, Limburg, Netherlands; Department of Research and Education, CIRO, Horn, Hornerheide, Netherlands

**Keywords:** *Palliative care*, *interprofessional*, *transitions*, *collaboration*, *multidisciplinary*, *interdisciplinary*, *home care*, *nursing home*

## Abstract

A qualitative study was conducted to examine the experiences of informal caregivers of persons with dementia pertaining interprofessional collaboration with and among healthcare professionals in home care (HC), nursing homes and during home to nursing home transitions in palliative care. Semi-structured interviews were performed with bereaved informal caregivers. Data were analysed using a critical realist approach. The two main themes that emerged were: (*1) Informal caregivers’ roles in interprofessional collaboration with healthcare professionals* and (*2) Informal caregivers’ perception of interprofessional collaboration among healthcare professionals*. Informal caregivers’ roles were identified in three collaboration processes: information exchange, care process and shared decision-making. Interprofessional collaboration among healthcare professionals was more perceptible on the collaboration outcome level (e.g. being up to date with the health status of the person with dementia; acting proactive, being adequate and consistent in the care process; and giving a warm welcome) than on the collaboration processes level (e.g. communicating and being involved in team processes). Our study revealed that intrinsic and extrinsic factors and interprofessional collaboration among healthcare professionals affected informal caregivers’ collaborative roles. In summary, our study showed that informal caregivers have important roles as team members in the continuity and quality of palliative care for persons with dementia.

## Introduction

Persons with dementia have complex care needs and problems involving cognitive, physical, behavioural, psychosocial and spiritual domains (Grand, Caspar, & Macdonald, 2011; Passos, Sequeira, & Fernandes, 2012; Perrar, Schmidt, Eisenmann, Cremer, & Voltz, 2015; Steen et al., 2014). As no curative treatments are currently foreseen for this life-limiting disease, a palliative care approach is paramount (van der Steen et al., 2016). One of the main components for optimal palliative dementia care comprises interprofessional collaboration (Davies et al., 2014; Fox et al., 2018; Steen et al., 2014). According to the World Health Organization, interprofessional collaboration is defined as *‘a situation when multiple health workers from different professional backgrounds work together with patients, families, caregivers and communities to deliver the highest quality of care.’* (WHO, 2010) Interprofessional collaboration results in positive outcomes for the person with dementia and family members (e.g. person-centred care, participation, empowerment and satisfaction), healthcare professionals (e.g. job satisfaction, performance and mental health) and the entire care process (e.g. effectiveness, efficiency, safety, quality and continuity of care) (D'Amour, Ferrada-Videla, San Martin Rodriguez, & Beaulieu, 2005; Körner et al., 2016; Stutsky & Laschinger, 2014). Within interprofessional collaboration, the person with dementia and informal caregivers (referring to families, close acquaintances or friends) are identified as crucial collaborative partners (Careau et al., 2015; D'Amour et al., 2005; McDonald & McCallin, 2010).

While persons with dementia retain their position in the centre of interprofessional collaboration, their ability to communicate their needs and wishes and make decisions independently decline as dementia progresses (Henriksen, Moholt, & Blix, 2020). Therefore, informal caregivers need to contribute to the relational autonomy (Lindeza, Rodrigues, Costa, Guerreiro, & Rosa, 2020) and person-centred care throughout the entire life journey of persons with dementia (Committee on Family Caregiving for Older Adults; Board on Health Care Services; Health and Medicine Division; National Academies of Sciences, 2016 Nov). Even though the majority of persons with dementia prefer to live at home (von Kutzleben, Schmid, Halek, Holle, & Bartholomeyczik, 2012), 75% to 95% of persons with dementia die in nursing homes. These home to nursing home transitions are often due to overburdened informal caregivers, and constitute one of the most common transitions in dementia care (Houttekier et al., 2010).

Several studies have explored interprofessional collaboration between informal caregivers of persons with dementia and healthcare professionals in home care (HC), in nursing homes and during nursing home transitions. These studies mainly showed that informal caregivers act as caregivers (White et al., 2018); provide person-centred information to stimulate tailored dementia care (Häikiö, Sagbakken, & Rugkåsa, 2020); make decisions regarding assessments and care plans (Dalgarno et al., 2021; Hughes, Woods, Algar-Skaife, Jelley, & Jones, 2019); facilitate continuity and management of care (Bunn et al., 2017); and contribute to the overall quality of life (Hughes et al., 2019) of persons with dementia in HC. Furthermore, in nursing homes, interprofessional collaboration between informal caregivers and nursing home staff is important as well due to their continued involvement in (Backhaus et al., 2020), for example, providing personal care (Häikiö et al., 2020; Roberts & Ishler, 2017); watching over the care process (Hoek et al., 2020); providing psychosocial support (Gaugler, 2005); and providing support in solving problems (Jang, 2020). Furthermore, collaboration with informal caregivers progresses during nursing home transitions (Groenvynck et al., 2021), as they are actively involved in making transition-related decisions (Garvelink et al., 2019) and transferring person-centred information (Graneheim, Johansson, & Lindgren, 2014; Pritty, De Boos, & Moghaddam, 2020). Garvelink et al. (2019) used an interprofessional collaboration model (Légaré et al., 2011) to describe the components important in shared decision-making concerning nursing home transitions such as informing and explaining the decision, identifying values and preferences and providing an overview of the feasible options (Garvelink et al., 2019).

However, to our knowledge, merely one study addressed the experiences of informal caregivers concerning both interprofessional collaboration together with and among healthcare professionals (Stephan, Möhler, Renom-Guiteras, & Meyer, 2015). Stephan et al. (2015) revealed that informal caregivers wish for one permanent contact person, notice that healthcare professional lack time to optimally collaborate with each other, and fill in the care gaps when they experience insufficient interprofessional collaboration among healthcare professionals. Hence, our study aimed to examine the experiences of informal caregivers of persons with dementia concerning both interprofessional collaboration together with healthcare professionals and among healthcare professionals themselves within HC, nursing homes and as well as during nursing home transitions.

## Methods

### Study design

This qualitative study is part of the larger Desired Dementia Care Towards End of Life (DEDICATED) research project executed in three Dutch care organizations (located in the southern region) offering HC and nursing home care. DEDICATED aims to improve the quality of palliative care for persons with dementia in HC, nursing homes and during nursing home transitions (AWO, 2017). Semi-structured in-depth interviews were done with bereaved informal caregivers of persons with dementia. This study followed the Consolidated criteria for Reporting Qualitative research (COREQ) (Tong, Sainsbury, & Craig, 2007) available in Supplement I.

### Recruitment of participants

Nurses from the three partner organizations of DEDICATED recruited participants using purposive sampling (Palinkas et al., 2015). The closest informal caregiver of a deceased person with dementia was eligible when their informal caregiver with dementia was 65 years of age or older, received care from one of the three care organizations and died between 6 weeks and 6 months prior to the inclusion period. Participants provided written informed consent prior to the interviews.

### Interview list

The interview questions exploring the themes ‘interprofessional collaboration’ and ‘transmural collaboration’ were distilled from a Delphi study defining optimal palliative care elements (Steen et al., 2014), a literature review identifying the needs of persons with dementia (Perrar et al., 2015), and the Dutch quality framework for palliative care (IKNL/Palliactief, 2017). Three researchers together with the research team of DEDICATED established preliminary questions. These questions were discussed with healthcare professionals from the three care organizations, patient representatives and experts in the field of dementia and palliative care in order to evaluate face validity. Subsequently, consensus with the research team of DEDICATED was reached and feasibility of the interview questions was evaluated through three pilot interviews with informal caregivers (Supplement II).

### Data collection

The interviews were conducted between February and July 2018 and took place either in the care organization or at the participants’ own home. Three female researchers performed the interviews in pairs (one guided the interview and the other one observed and asked follow-up questions). Data were audio recorded and transcribed according to the clean-read verbatim method (Supplement III).

### Data analysis

IBM SPSS statistics version 25 was used to carry out the descriptive analyses. For the qualitative analysis, NVIVO version 11 was used, and a critical realist approach was employed (Fletcher, 2017), underpinned by a transformative paradigm (Ben Salah, 2015). The analysis was based on two collaboration levels (collaboration with and collaboration among healthcare professionals), and guided by existing knowledge about informal caregiver involvement and collaboration experiences (Astrid Stephan et al., 2015). The coding procedure was an iterative process combining inductive, deductive (DeCuir-Gunby, Marshall, & McCulloch, 2011), abduction and retroduction methods (Fletcher, 2017). Details about the data analysis procedure are described in Supplement IV, and the coding tree is provided in Supplement V.

## Findings

**Figure 1.**
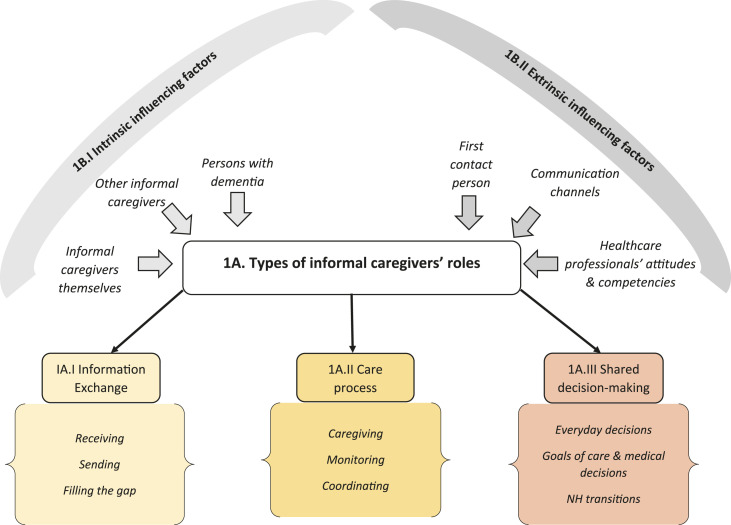
*Analysis scheme of Theme 1. Informal caregivers’ roles in interprofessional collaboration with healthcare professionals*.

**Figure 2.**
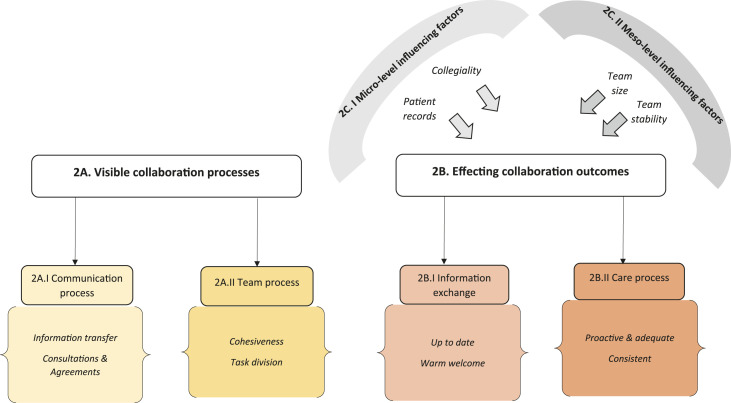
*Analysis scheme of Theme 2. Informal caregivers’ perception of interprofessional collaboration among healthcare professionals*.

In total, 32 informal caregivers participated (response rate = 71.1%). 13 candidates refused participation because they were either not interested or found the topic too sensitive. The majority of informal caregivers were female, had a mean age of 62.0 years (SD = 9.3) and were children of the deceased person with dementia. Most persons with dementia were female, had a mean age of 86.6 years (SD = 6.3) and died in a nursing home (75%) (Table 1). The mean interview time was 92.0 min (SD = 17.6 and IQR = 27.8). The findings were divided into two main themes related to the study objectives (Figures 1 and 2).

**Table 1.** *Characteristics of informal caregivers and persons with dementia*.

	*N* = 32
*Demographic characteristics of informal caregivers*	
Age, mean (range)	62 (44–87)
Female gender, number (%)	22 (68.8)
Education level, number (%)	
Preparatory secondary vocational education	9 (28.2)
Senior secondary vocational education & training	11 (34.4)
Senior general secondary education and university preparatory education	4 (12.5)
Applied science or university education	8 (25.0)
*Relationship between informal caregivers and persons with dementia*	
Type of informal caregivers, number (%)	
Spouses (married and divorced)	4 (12.5)
Children (biological, step and in-law)	23 (71.9)
Siblings	1 (3.1)
Cousins (biological and in-law)	3 (9.4)
Friends	1 (3.1)
*Demographic characteristics of the persons with dementia*	
Age, mean (range)	87 (70–97)
Female gender, number (%)	23 (71.9)
*Care characteristics of persons with dementia*	
Type of care setting in which the person with dementia died, number (%)	
Home care setting	4 (12.5)
Nursing home setting	25 (78.1)
Hospital	3 (9.4)
Duration of receiving home care, number (%)	8 (25.0)
0–1 years	2 (6.2)
1–2 years	3 (9.4)
2–5 years	3 (9.4)
Duration of receiving nursing home, number (%)	24 (75.0)
0–1 years	9 (28.1)
1–2 years	9 (28.1)
2–5 years	6 (18.8)

### Theme 1. Informal caregivers’ roles in interprofessional collaboration with healthcare professionals

We identified three different types of processes in which informal caregivers had collaborative roles in interprofessional collaboration with healthcare professionals and two types of factors influencing these roles (Figure 1). The findings contain key quotes of informal caregivers, which are described using pseudonyms (Table 2), and describe key differences of informal caregivers’ collaborative roles in HC, nursing homes and during nursing home transitions (Table 3).

**Table 2.** *Pseudonyms of informal caregivers*.

Pseudonym	Age	Relationship with person with dementia	Healthcare setting
*Mr. Deitman*	63	Son of a woman with dementia	Nursing Home
*Mrs. Fendaal*	59	Daughter of a woman with dementia	Nursing Home
*Mrs. Pike*	58	Daughter of a woman with dementia	Home Care
*Mrs. Glewen*	56	Daughter-in-law of a man with dementia	Home Care
*Mrs. Camping*	59	Daughter of a man with dementia	Nursing Home
*Mrs. Covers*	60	Daughter of a woman with dementia	Nursing Home
*Mrs. Pas*	55	Stepdaughter of a woman with dementia	Nursing Home
*Mrs. Mink*	64	Niece by marriage of a woman with dementia	Nursing Home
*Mr. King*	44	Son of a woman with dementia	Nursing Home
*Mrs. Kappas*	74	Friend of a woman with dementia	Nursing Home
*Mrs. Fink*	61	Daughter of a man with dementia	Nursing Home
*Mrs. Veldboom*	53	Daughter of a woman with dementia	Nursing Home
*Mr. Neckers*	72	Ex-husband of a woman with dementia	Nursing Home
*Mrs. Hengfeld*	67	Daughter of a woman with dementia	Nursing Home
*Mrs. Wordes*	61	Daughter of woman with dementia	Nursing Home
*Mr. Wellhouse*	63	Son of a woman with dementia	Nursing Home
*Mrs. Rordink*	53	Daughter of a woman with dementia	Nursing Home
*Mrs. Armslow*	62	Daughter of a woman with dementia	Nursing Home
*Mr. Peters*	85	Brother of a woman with dementia	Nursing Home
*Mrs. Fox*	75	Wife of a man with dementia	Nursing Home
*Mr. Jackson*	53	Son of a man with dementia	Home Care
*Mrs. Johnson*	62	Daughter of a woman with dementia	Nursing Home
*Mr. Garrison*	61	Son-in-law of a man with dementia	Nursing Home
*Mr. Damcott*	51	Son-in-law of a man with dementia of	Home Care
*Mrs. Miller*	54	Niece of a man with dementia	Home Care
*Mrs. Brewer*	65	Wife of a man with dementia	Home Care
*Mr. Lomis*	63	Son of a man with dementia	Home Care
*Mrs. Williams*	87	Wife of a man with dementia	Home Care
*Mrs. Smith*	72	Daughter of a woman with dementia	Nursing Home
*Mrs. Robinson*	55	Daughter of a woman with dementia	Nursing Home
*Mrs. Fisher*	56	Daughter-in-law of a woman with dementia	Nursing Home
*Mrs. Thatcher*	60	Niece of a woman with dementia	Nursing Home

**Table 3.** *Key differences of informal caregivers’ roles in interprofessional collaboration with healthcare professionals between HC, nursing home and nursing home transitions*.

Care Transition	Informal caregivers’ roles in interprofessional collaboration together with healthcare professionals
*HC*	*More focus on:* • *Caregiving & monitoring* • *Making every day, goals of care and medical decisions* • *Coordinating care at home (seeking information and support and executing care arrangements)* • *Anticipating on* *nursing home* *transitions* • *Intermediary among healthcare professionals*
Nursing home *transition*	*More focus on:* • *Making* *nursing home* *transitions decisions* • *Coordinating the* nursing home *transition* • *Filling the information gap* • *Providing personal information*
Nursing home	*More focus on:* • *Asking for information about the* *nursing home* *life and health situation of their relative* • *Filling the information gap* • *Additional caregiving, monitoring & coordinating* • *Making every day, goals of care and medical decisions*

#### Types of informal caregivers’ roles

*Information exchange*: Receiving, Sending and Filling the gap

##### Receiving

All informal caregivers needed information from healthcare professionals about dementia, palliative care, end-of-life care and the progression of the disease, and requested regular updates about the current health status, medication list, care process and overall wellbeing of the person with dementia to understand the current situation and anticipate on the future. *‘After she passed away, they explained us that it is normal that her eyes were open. But we did not know that, so at the time of her death we were worried because we thought that she was still conscious.’ (Mrs Rordink)* Besides, informal caregivers from HC also required practical information to execute care arrangements. *‘That support group guided us. Otherwise, you do not know where to be. Especially, when my dad could not wash himself any more and more care provision was needed.’ (Mr. Lomis)* Next, informal caregivers in nursing homes preferred general information about the nursing home life. Informal caregivers indicated the importance to receive information about the general care process or care experience of the person with dementia. In dementia care, informal caregivers required information from healthcare professionals, as persons with dementia often displayed recall issues or were not able to communicate what they experienced. However, about half of the informal caregivers expressed a lack of receiving adequate or timely information. In response, informal caregivers either proactively asked healthcare professionals for information, searched for information on the internet or approached their informal network to gain information. *‘I attached a plastic bag to the wheelchair and put a notebook in it, so that they could write down what my dad did at day-care.’ (Mr. Jackson)*

##### Sending

All informal caregivers provided personal information about the person with dementia to healthcare professionals. This information included their life history, likes and dislikes in daily life, daily habits, hobbies, care needs and capabilities in daily care, preferences concerning social and religious activities and end-of-life wishes. Informal caregivers explained that taking into account personal information provided by them satisfied persons with dementia, because they were not able to express these themselves. Moreover, it could also benefit healthcare professionals as it smoothens cooperation with the person with dementia and might prevent escalations: ‘*I saw the nurse and my aunty slapping each other. I told the nurses many times that my aunty wanted to wash herself, because she was afraid that someone would touch her.*’ *(Mrs. Thatcher)* Personal information was often transferred through verbal communication; however, two informal caregivers were also asked to document this information in a life story book together with healthcare professionals. Most informal caregivers were asked to provide personal information, especially during the intake conversation at the nursing home admission.

##### Filling the gap

About half of the informal caregivers repeated information to, actively relayed information between or functioned as an information intermediary among healthcare professionals. Nine informal caregivers in nursing homes noticed that the care needs and preferences of persons with dementia were not taken into account. In response to this observation, they either repeated information *(‘I decided to write it on the board but even then, they still did not put her hearing aids*.’ *(Mrs. Wordes))*, or actively relayed information *(*‘*If I had my iPad with me, I could have showed you how many emails I sent to them due to lack of communication among them.’ (Mrs. Thatcher))*. Furthermore, seven informal caregivers described that they filled the information gap. Of these seven informal caregivers, five informal caregivers repeated information and two informal caregivers actively related information during nursing home transitions: ‘*It was already an emotional situation and I also had to tell them twice to hand off the patient file to the nursing home. I really felt overburdened.*’ *(Mr. Deitman)* However, informal caregivers in HC functioned more as an intermediary among healthcare professionals, but did not experience this as exhausting, as they did not have to repeat information, but namely pass through information. Moreover, in HC, healthcare professionals themselves transferred information through the patient file, which they left at the residence of the person with dementia.

*Care process: Caregiving, Monitoring and Coordinating*

##### Caregiving

Informal caregivers in HC performed general care tasks such as basic care tasks (i.e. meal preparation, feeding, toilet guidance and skin care) and household tasks. Especially, their contribution to managing medication, financial tasks and appointments of the person with dementia were important due to the cognitive decline. In HC, most informal caregivers expressed feeling part of the team, because they had close interactions with healthcare professionals (often district nurses), directly accessed information (through reading the patient file at home) and divided care tasks. *‘The district nurses and I form one team. When something happened or changed, we always discussed it together.’ (Mrs. Brewer)* In nursing homes, most informal caregivers did not feel part of the team, because they had to chase the first contact persons and other healthcare professionals to exchange information. Further, even though informal caregivers in nursing homes performed activities to make the person with dementia feel at home such as playing music, walking outside, eating out at restaurants and visiting with other informal caregivers, less than a quarter performed additional caregiving roles in response to inadequate care provision. *‘My aunty had a bladder infection and sometimes had to wait half an hour for a nurse to go the toilet, so that is why I helped her myself when I was there.’ (Mrs. Mink)*

##### Monitoring

Monitoring refers to verifying whether healthcare professionals took into account the personal needs and preferences of persons with dementia, reviewed their care provision and signalled changes in their health situation. *‘It took me half a year to ensure that she received clean clothes every day.’ (Mrs. Rordink)* Three quarters of the informal caregivers in nursing homes showed additional monitoring roles such as noticing when care tasks were not executed and responding to it by communicating their dissatisfaction and/or reactive caregiving. On top of that, informal caregivers signalled changes themselves when healthcare professionals did not identify those changes. ‘*The nurses did not find it necessary to call the physician, but I saw that there was something wrong with her eye, and indeed when the physician came he diagnosed stroke.*’ *(Mrs. Covers)* Moreover, when healthcare professionals did not act quickly upon changes, informal caregivers themselves took control. ‘*When I saw my mom in pain and asked for paracetamol, they said they have to ask the physician. If she had pain on Tuesday, she had to wait one week, because the physician was only available on Monday. Therefore, I secretly gave her paracetamol myself.*’ *(Mrs. Robinson)*

##### Coordinating

Coordinating covers filling the information gap, performing care arrangements or actively approaching healthcare professionals and care organizations to ensure that care is person-centred, safe and continuous. Informal caregivers in HC ensured adequate medication supply; arranged sufficient HC support; requested supporting materials to live at home as long as possible; involved necessary healthcare professionals; functioned as an intermediary; organized transport for appointments; and anticipated on nursing home transitions. ‘*The district nurse told me that my dad has been removed from the client list and she was not allowed to do anything with the extension approval from the Social Support Act. Therefore, I have been alone with my dad that whole weekend.* ’*(Mr. Lomis)* During nursing home transitions, informal caregivers coordinated the transition through initiating the transition; looking for the appropriate nursing home; contacting healthcare professionals from the nursing home; applying to the Dutch care needs assessment centre to receive permission for the nursing home transition; clearing and selling the house; arranging transport to the nursing home; and furnishing the nursing home room. Moreover, two informal caregivers coordinated the information transfer between HC and nursing home, and three informal caregivers applied the Care and Compulsion act to execute an involuntary nursing home transition. In nursing homes, informal caregivers coordinated through filling the information gap, contacted healthcare professionals when no responsibility was taken to monitor care and follow the rules, and emphasized the presence of supporting materials (i.e. motion sensors, pressure ulcer mattress, wheel chair cushions or safety belts). *‘I saw hydration protocols in her room which were not filled in. I showed them and asked who was then responsible for doing that and taking care of her during mealtimes. One day her soup was cold because she waited 1.5 h but no one came to feed her so I asked whether they took into account the Hazard Analysis Critical Control Points rules.’ (Mrs. Pas)* In addition, most informal caregivers felt rushed and emotional when making the nursing home transition decision and transporting the person with dementia to the nursing home, and therefore wished support from healthcare professionals during this process. After the death of the person with dementia, informal caregivers (in HC and nursing homes) coordinated the funeral such as contacting the mortician and arranging finances.

*Shared decision-making: Everyday decisions, Goals of care & medical decisions* and *nursing home transitions*

##### Everyday decisions

All informal caregivers (in HC and nursing homes) were involved in making everyday decisions about personal care and supporting materials, as persons with dementia were not able to make these decisions. Informal caregivers also developed methods to cope with distressed behaviours of persons with dementia and made sure that they maintained their activities and remained connected with their familiar environment. Informal caregivers (mostly in nursing homes) pointed out that healthcare professionals did not always align with them when making everyday decisions. *‘I came to know that the recreational therapist did reminiscence through painting with my stepmom. My stepmom painted a very intense life event. Afterwards, she got delirium and relived that event in her dreams. I was so angry about that, because it was not pre-discussed with me.’ (Mrs. Pas)*

##### Goals of care & medical decisions

The majority of informal caregivers (in HC and nursing homes) focussed on comfort, enjoyment and satisfaction in life when communicating personal preferences of the person with dementia. They also emphasized on relief of suffering and prevention of burdensome treatments when setting goals of care and making medical decisions for the person with dementia. Medical decisions about administration of opioids, hospital admissions, medication, food and fluid administration, palliative sedation and resuscitations were made together. Healthcare professionals initiated discussions to ask informal caregivers what they wanted and what the person with dementia would have preferred. In this way, they both tried to reach consensus and make the best suitable and shared decision for the person with dementia. ‘*The physician proposed to give our mother antibiotics, but we did not agree, discussed this together with the physician and decided to stop with the medication.*’ *(Mrs. Hengfeld)*

##### Nursing home transitions

Eleven transitions occurred from care homes or sheltered homes to nursing homes, ten transitions took place from home to nursing homes and three transitions from somatic to psychogeriatric nursing home wards. In addition, four persons with dementia living at home or in a care home were not able to return home after their stay at the hospital or revalidation centre, and therefore had to move directly to a nursing home. More than half of the informal caregivers initiated nursing home transition conversations, as they noticed the increasing physical and cognitive decline of the person with dementia, safety issues and burden of other informal caregivers and themselves. Most of them did not feel supported when making the transition decision, as healthcare professionals did not anticipate with and/or guide them. Two informal caregivers even had to persuade their physician of their inability to cope with increasing care demands and to request support towards a nursing home transition. Even though most informal caregivers made the transition decision, some informal caregivers afterwards felt supported by healthcare professionals as they had the opportunity to carry out conversations about institutionalization during family meetings or visits. In all cases, informal caregivers were involved in choosing a nursing home and paid attention to the following preferences: appropriate ambiance, home-like feeling, available activities, familiar environment and short travel distance to their home. Some informal caregivers mentioned that they preferred a nursing home in which residents were in the similar stage of dementia as the person with dementia. They explained that when the other residents were in a more advanced stage of dementia in comparison to the person with dementia, they saw the person with dementia declining faster.

#### Factors influencing informal caregivers’ roles

*Intrinsic factors*: Informal caregivers’ themselves, Other informal caregivers and Persons with dementia

The type of and extent of informal caregivers’ collaborative roles, in the first place, depended on informal caregivers themselves (i.e. willing to be involved, being assertive and having knowledge of or a previous experience in the care process). Second, when other informal caregivers were involved they felt supported. About one-third of the informal caregivers had a more active role because they were healthcare professionals themselves, worked in the healthcare sector or/and had informal caregivers who were healthcare professionals. Finally, the capabilities of persons with dementia to express themselves and their place of residence also influenced informal caregivers’ roles.

*Extrinsic factors*: First contact person, Communication channels and Healthcare professionals’ attitudes & competencies

The majority emphasized the importance of having a first contact person, as informal caregivers find it important to know who to approach to exchange information, coordinate care and seek support. First contact persons were often nurses (in HC and nursing home), case managers dementia (in HC) or physicians (in HC). Communication channels used in HC and nursing homes to exchange information were phone calls, e-mails (usually to inform, update or check) and one-on-one conversations (mostly with first contact person or physician to inform, discuss or decide). In addition, informal caregivers in HC had patient files (usually to document actions and changes and transfer information), while in nursing homes informal caregivers were able to participate with multidisciplinary team meetings (usually twice a year to discuss overall health, care plans, problems and complaints). *‘I would have preferred a second intake conversation after the nursing home transition together with the multidisciplinary team in order to have everyone on the same page, directly from the start. Additionally, I would organize these meetings to conduct mid-term evaluations to ask how we experienced the care process and discuss their performance.’ (Mrs. Fink).* Informal caregivers often requested communication with the first contact person and the physician. However, in nursing homes, their first contact person was not always available or constantly changed and most physicians were physically present only once a week.

When healthcare professionals had a proactive attitude and adequate competencies, informal caregivers felt more part of the team and showed fewer reactive roles such as asking for information; filling the information gap; and additional caregiving; monitoring; and coordinating. According to informal caregivers, healthcare professionals’ proactive attitudes comprised exchanging information with informal caregivers; taking into account preferences and needs of the person with dementia; having attention for the person with dementia; listening to informal caregivers; involving informal caregivers in decision-making processes; exchanging information with other healthcare professionals; and seeking for solutions. Adequate competencies of healthcare professionals were described as having knowledge; signalling changes; having communication skills; coping with distressed behaviours of the person with dementia; and acting proactively and adequately in the care process.

### Theme 2. Informal caregivers’ perception of interprofessional collaboration among healthcare professionals

Informal caregivers had little insight into the collaboration processes among healthcare professionals, but could notice the collaboration outcomes that effected their informal caregiver and their collaborative roles (Figure 2)*.* Informal caregivers additionally described two factors influencing these collaboration outcomes.

#### Visible collaboration processes

##### *Communication process*: Information transfer and Consultations & Agreements

Informal caregivers were able to report to healthcare professionals that were closely involved in the care process (Table 4). In most cases, nurses, physicians, volunteers, physical therapists, occupational therapists and volunteers were involved. The majority of informal caregivers assumed that healthcare professionals communicated with each other (via phone calls and meetings) to transfer information, consult with each other and develop collaboration agreements. The communication process among healthcare professionals was visible to informal caregivers in HC and nursing home, respectively, when they wrote in the patient files and performed home visits together, and called each other or talked with each in front of them. During nursing home transitions, some informal caregivers were aware of the information transfer. *‘The general practitioner and elderly care physician from the nursing home exchanged information. After the transition, the general practitioner also called me.’ (Mrs. Pas)*

**Table 4.** *Informal caregivers’ perception of all involved healthcare professionals*.

Healthcare setting	Nurses	Physician	CM	CA	V	RT	PT	OT	Psy	RD	Others*
HC	All	All	Some	-	All	Half	Half	Half	-	One	One
Nursing homes	All	All	-	-	Major	Major	Half	Half	Half	Some	Onethird
Nursing home transitions	Some	Some	One	Some	-	-	-	-	-	-	-

The quantifiers ‘one third’, ‘some’, ‘half’, ‘major.’ and ‘all’ are used to indicate how many informal caregivers reported the involvement of the HCP.

Abbreviations: Major. = Majority; CM = Case manager dementia; CA = Client advisor from the nursing home; V = Volunteers; RT = Recreational therapist; PT = Physical therapist; OT = Occupational therapist; Psy. = Psychologist; RD = Registered dietician.

Others* = One social worker in HC; three speech therapists, one podiatrist, two dentists and two spiritual caregivers in nursing homes.

##### *Team process*: Cohesiveness and Task division

Seven informal caregivers (in nursing homes) observed and accentuated that team cohesiveness, which refers to the feeling that all healthcare professionals are equal to each other, belong to one team and work towards the same goal, is important in the collaboration process. *‘Those who carried out household tasks and those who executed care tasks did not form one team. Even though it was clearly written down in the file to close the curtains, they did not do it. Due to this, she still fell two to three times afterwards (while trying to do it herself).’ (Mrs. Thatcher)* Apart from the feeling of working together, all informal caregivers knew their distinct professional tasks and assumed that healthcare professionals did divide their tasks as team members, but did not know or want to know the details. A few informal caregivers noticed differences among healthcare professionals in terms of eligibility to perform certain care tasks. *The nurse said: ‘Yes, I am level four and officially allowed to administer morphine by myself, but during the night I am level three, because the organization wants to save money. Then a second nurse has to be there too. Those are the rules.’ (Mr. Damcott)*

#### Effecting collaboration outcomes

##### *Information exchange*: Up to date and Warm welcome

One third of the informal caregivers in nursing homes and half of the informal caregivers during nursing home transitions experienced that healthcare professionals were not up to date with the current health situation of the person with dementia, and in reaction to this, informal caregivers filled the information gap. *‘I was bewildered when the nurses did not know that we stopped with the antibiotics, so I tried to keep up the communication with them’. (Mrs. Armslow)* Half of the informal caregivers mentioned that healthcare professionals were not aware of the complete health situation, needs and preferences of the person with dementia. They provided (additional) personal details about the person with dementia during the intake conversation at the nursing home admission. Further, seven informal caregivers were actively involved in the handoff of patient information (e.g. sharing patient record or personal details). ‘*I had to repeat a lot of information. The medication list was also incorrect. I do not understand why they did not involve me during the handoff of patient information?*’*(Mrs. Wordes)* However, according to most informal caregivers, nursing home healthcare professionals provided a warm welcome to the person with dementia when they prepared sweets and drinks, carried out intake conversations and provided a guided tour. *‘We received a warm welcome, it was great, there was a table ready, there was cake present, and it all gave us a celebratory feeling.’ (Mrs. Fink)*

##### *Care process*: Proactive & adequate and Consistent

For the most part, informal caregivers in nursing homes experienced that healthcare professionals acted proactive and adequate in the care process. In nursing homes, some informal caregivers experienced communication delays between nurses and physicians, and therefore expressed the need for shorter communication lines between them. In HC, informal caregivers either called the physicians themselves or asked the nurses to call a physician. Moreover, one third of the informal caregivers noticed that not all healthcare professionals executed their care tasks in the same way. One informal caregiver described the consequence of inconsistent handling of opioid administration: *‘They were allowed to use the fentanyl nasal spray six times per day, but sometimes they only sprayed two or three times while my mom indicated that she experienced pain.’ (Mrs. Johnson)*

#### Factors influencing collaboration outcomes

##### *Micro-level factors*: Patients records and Collegiality

Eight informal caregivers (in nursing homes) reasoned that when healthcare professionals were not up to date with the health situation of the person with dementia or did not consistently execute the care tasks, they probably did not read the patient record themselves (individual responsibility) and/or did not inform each other about the changes (collegial responsibility). *‘A substitute nurse called me to ask whether we could bring a razor, while I already told them and it was also written in the file that she did not want to shave. Therefore, we already arranged a beautician for her.’ (Mrs. Fendaal)*

##### *Meso-level factors*: Team size and Team stability

The majority of informal caregivers (in nursing homes) had the feeling that healthcare professionals probably did not have time to transfer information, could not optimally inform all healthcare professionals and therefore could not always act proactive and adequate in the care process. They also mentioned the lack of staff and the continuous change of team compositions that influenced staff performance and the way they provided personal attention to the person with dementia, which is a relevant aspect of dementia care. *‘They did not have time to give personal attention, because there were not enough nurses.’ (Mr. Neckers)* Furthermore, informal caregivers noticed high staff turnovers and many different or temporary substitutes, volunteers and interns, which could make it difficult to build a strong and stable team.

## Discussion

This is the first study that investigated the roles of informal caregivers in interprofessional collaboration as well as their perception of interprofessional collaboration among healthcare professionals in dementia care. Our findings showed that informal caregivers’ main roles in interprofessional collaboration with healthcare professionals were exchanging information, acting in the care process and making shared decisions. Further, we revealed intrinsic factors (related to informal caregivers themselves, other informal caregivers and the persons with dementia) and extrinsic factors (related to the healthcare professionals and the care organizations) which influence to which magnitude and which (additional) roles informal caregivers performed. Moreover, next to these factors, interprofessional collaboration among healthcare professionals, which informal caregivers noticed on the level of collaboration process and outcomes, affected informal caregivers’ collaborative roles. This illuminates that interprofessional collaboration among healthcare professionals and interprofessional collaboration between informal caregivers and healthcare professionals exhibit a mutual dependence, as they influence with each other.

In concordance with our findings, previous research showed that informal caregivers need general information about dementia (Doornebosch, Smaling, & Achterberg, 2022; White et al., 2018) and information about and support during nursing home transitions (Groenvynck et al., 2021). Further, in HC, they face diverse healthcare professionals and care organizations which make it difficult for informal caregivers to coordinate the care process for persons with dementia (Bieber et al., 2018; Lethin, Hallberg, Karlsson, & Janlöv, 2016; Nordtug et al., 2021; White et al., 2018). Correspondingly, Häikiö et al. 2020 illustrated that informal caregivers of persons with dementia used assertive strategies in interprofessional collaboration with healthcare professionals such as alerting healthcare professionals, using social relationships and filling complaints (Häikiö et al., 2020).

The importance of family involvement has been emphasized earlier in general care for older persons (Bélanger, Desmartis, & Coulombe, 2018; Van Houdt, De Lepeleire, Driessche, Thijs, & Buntinx, 2011) and general palliative care (Hudson, Quinn, O'Hanlon, & Aranda, 2008; Neto & Trindade, 2007) (Górska, Forsyth, Prior, Irvine, & Haughey, 2016). In dementia care, informal caregivers act as person-centred information sources (Ponnala, Block, Lingg, Kind, & Werner, 2020), advocators (Bunn et al., 2017) and decision-makers (Livingston et al., 2010), which is especially important to provide person-centred (Reid & Chappell, 2017) due to the cognitive decline and communication difficulties of persons with dementia (Hinton et al., 2007). Moreover, through expressing the personal wishes and needs of the persons with dementia, informal caregivers contribute to ensuring comfort, enjoyment and satisfaction (Fox et al., 2018), and support healthcare professionals in understanding distressed behaviours of persons with dementia (Hughes et al., 2019). We have described in our findings that by sharing personal information and needs, family members may prevent escalations due to challenging behaviours. Existing literature confirms the relation between unmet needs and challenging behaviour in dementia (Duxbury, Pulsford, Hadi, & Sykes, 2012; Schnelli, Karrer, Mayer, & Zeller, 2020). Furthermore, Huis in het Veld et al. (2016) showed that family members could also contribute to managing challenging behaviours through sharing the self-management strategies they have used themselves under which calming down and stimulating their informal caregiver (Huis in het Veld et al., 2016).

Informal caregivers’ continuous involvement secures informational continuity of PC (Haggerty et al., 2003), which is indispensable during the care process in HC, nursing home and peculiarly during nursing home transitions. Without optimal coordination between the care settings (e.g. referrals, collaboration and information transfer) (Ashbourne, Boscart, Meyer, Tong, & Stolee, 2021), these nursing home transitions are susceptible to adverse outcomes such as miscommunication and medication errors (Callahan et al., 2012). Known effective components for an optimal nursing home transition for persons with dementia include: (1) shared decision-making; (2) preparing and supporting the person with dementia and their informal caregivers for the nursing home transition; (3) collecting all information of the person with dementia; (4) transferring information between the care settings; (5) using this information to prepare the welcome of the person with dementia and their informal caregivers; (6) performing follow-up by healthcare professionals from HC; and (7) assisting in adjusting to a new environment by healthcare professionals from nursing home (Ashbourne et al., 2021; Coleman, 2003; Garvelink et al., 2019; Groenvynck et al., 2021).

As informal caregivers are the ‘constant factor’ throughout the entire transition process (Feinberg, 2012), it is prominent to incorporate their views from the start through an interprofessional shared decision-making process (Legare et al., 2014; Miller, Whitlatch, & Lyons, 2016). Little is known about shared decision-making regarding nursing home transitions in dementia care (Garvelink et al., 2019). However, similar to the findings of Garvelink et al. (2019), we found that most informal caregivers proactively proposed the nursing home transition decision, convinced healthcare professionals of the importance of the nursing home transition and acted more as autonomous decision makers during nursing home transitions (Garvelink et al., 2019). Moreover, during information transfer between care settings, informal caregivers in our study mentioned that they play an immense role in making sure that accurate person-related information and the correct medication list is transferred. Likewise, two previous studies have shown that family involvement could prevent medication errors that frequently occur during care transitions in dementia care (Deeks, Cooper, Draper, Kurrle, & Gibson, 2015; Taylor, Østbye, Langa, Weir, & Plassman, 2009). Furthermore, we have shown that family members mostly wished for and selected a nursing home with a home-like environment in which persons with dementia have the opportunity to perform meaningful activities. This is known to facilitate the adjustment process to the nursing home (Sury, Burns, & Brodaty, 2013), which is crucial in dementia care (Førsund et al., 2018) since these transitions can lead to additional disorientation and agitated behaviour in persons with dementia (Aminzadeh, Dalziel, Molnar, & Garcia, 2009; Rognstad et al., 2020). On top of that, the care preferences mentioned by the informal caregivers in our study, under which providing comfort care and preventing burdensome treatments, suit the principles of optimal palliative dementia care (Steen et al., 2014).

With respect to the collaborative needs of informal caregivers, we highlighted the need of a first contact person on behalf of the professional care team for the family. Within the interprofessional collaboration model described by Légaré et al. (2011), which focuses on general healthcare, a first contact person for family is recommended (Légaré et al., 2011). This HCP could identify the problem or challenge and can support decision-making. Specifically, in dementia care, the contact person could take the responsibility to guide the family, inform the family, manage services, facilitate communication with other healthcare professionals and could facilitate overall collaboration between family and healthcare professionals (Afram, 2015; Lethin et al., 2016; Stephan et al., 2015; Astrid Stephan et al., 2015). Moreover, our finding that many informal caregivers lacked support from healthcare professionals during nursing home transitions in dementia care (for example, when making transition-related decisions) is supported by other studies (Hanssen et al., 2021; Lord, Livingston, Robertson, & Cooper, 2016). Next, adopting a proactive attitude as a healthcare professional in providing timely information to informal caregivers about dementia, palliative and end of life care to informal caregivers (Goossens, Sevenants, Declercq, & Van Audenhove, 2020; White et al., 2018) and anticipating with informal caregivers on, for example, future transitions (Ashbourne et al., 2021; Lethin et al., 2016) are essential to enhance interprofessional shared decision-making.

Even though informal caregivers from our study did not express the need to clarify roles with healthcare professionals, existing literature emphasized the importance of role clarification and negotiation (Wittenberg, Kwekkeboom, Staaks, Verhoeff, & de Boer, 2018). Indeed, role negotiation is conducive since healthcare professionals and informal caregivers complement each other (Häikiö et al., 2020). Furthermore, role clarification can decrease reduce informal caregivers’ burden (Wittenberg et al., 2018) and role conflicts between informal caregivers and healthcare professionals (Bowers, 1988). Especially, during nursing home transitions role clarification is required (Bucknall et al., 2016), as informal caregivers may adopt their roles (Graneheim et al., 2014) from focussing more on housekeeping (Brodaty & Donkin, 2009), personal care and vigilance (Ponnala et al., 2020) in HC to emphasizing attention on advocating (Prince, Prina, & Guerchet, 2013), decision-making and additional monitoring in nursing homes (Hoek et al., 2020). Apart from identifying and discussing the types of roles, conversing about the participation level (Thompson, 2007) is also part of clarifying informal caregivers’ position within interprofessional collaboration. Besides talking about the participation level, we recommend healthcare professionals to continuously monitor (Galvin, Valois, & Zweig, 2014) and regularly evaluate the interprofessional collaboration with informal caregivers (Careau, Bainbridge, Steinberg, & Lovato, 2016), and request informal caregivers for feedback about the outcomes of interprofessional collaboration among healthcare professionals. This process could pinpoint the additional collaborative roles of informal caregivers, which they perform in response to the lack of responsibility of healthcare professionals or adequacy interprofessional collaboration among healthcare professionals (Jarrett, 2009; Astrid Stephan et al., 2015).

## Methodological considerations

In our study, strengths include the inclusion of informal caregivers with experience of interprofessional collaboration in HC, nursing homes and during nursing home transitions. Further, different researchers were involved in data collection and analysis, investigator triangulation took place (through an independent coding procedure and consultation meetings with the DEDICATED research team), and data saturation was achieved. Moreover, even though selection bias might have occurred as the recruiters (nurses) could have selected candidates they frequently saw or which they were familiar with, our study population was diverse (i.e. different types of informal caregivers, representative mix of man and woman and diverse educational backgrounds). Nevertheless, we only interviewed one informal caregiver per person with dementia. This informal caregiver was the closest involved informal caregiver. However, in general more informal caregivers surround a person with dementia. Of course, collaboration with all involved informal caregivers is relevant, but this was not covered in our study. In addition, we did not examine the perspectives of the involved healthcare professionals to compare the collaborative experiences from both sides. Finally, Dutch cultural and health care aspects may influence the generalizability of the outcomes.

## Conclusion

Our findings imply that informal caregivers in fact are key team members in palliative dementia care. We have shown that their collaborative roles could shift depending on which setting the person with dementia resides. In HC, informal caregivers’ roles focussed more on personal caregiving, coordinating care at home and anticipating on possible nursing home transitions for the person with dementia. During nursing home transitions, they felt that they were initiating, coordinating, filling the information gap and providing personal information about the person with dementia. In nursing homes, informal caregivers emphasized on asking information about the person with dementia, filling the information gap and performing additional caregiving, monitoring and coordinating roles to ensure person-centred care for the person with dementia. Moreover, we revealed that interprofessional collaboration among healthcare professionals could affect informal caregivers by urging them to take additional collaborative roles. Therefore, we recommend healthcare professionals to discuss the collaborative roles with and evaluate the interprofessional collaboration with informal caregivers. In conclusion, healthcare professionals should take into account different roles of informal caregivers in interprofessional collaboration. This might optimize the continuity and quality of palliative care in dementia.

## Supplemental Material

Supplemental Material - Interprofessional collaboration in palliative dementia care through the eyes of informal caregivers Click here for additional data file. Supplemental Material for Interprofessional collaboration in palliative dementia care through the eyes of informal caregivers by Chandni Khemai, Judith M Meijers, Irma Mujezinovic, Sascha R Bolt, Sabine Pieters, Albine Moser, Jos M G A Schols and Daisy J A Janssen in Dementia
