# Supplementary material for: Interprofessional collaboration in palliative dementia care through the eyes of informal caregivers
Source: Dementia (London). 2022 May 10;21(6):1890–913. doi: 10.1177/14713012221098259 (PMC9301172; doi:10.1177/14713012221098259)
Supplement: Supplemental Material - Interprofessional collaboration in palliative dementia care through the eyes of informal caregivers [file sj-pdf-1-dem-10.1177_14713012221098259.pdf]

Supplement I: Consolidated criteria for reporting qualitative studies (COREQ): 32-item checklist

Developed from:
Tong A, Sainsbury P, Craig J. Consolidated criteria for reporting qualitative research (COREQ): a 32-item checklist for interviews and focus groups. International Journal for Quality in Health Care. 2007. Volume 19, Number 6: pp. 349 – 357

No. Item	Guide questions/description	Reported on Page #
**Domain 1: Research team and reflexivity**		
Personal Characteristics		
1. Interviewer/facilitator	Which author/s conducted the interview or focus group?	Page 7
2. Credentials	What were the researcher's credentials? E.g. PhD, MD	Supplement III
3. Occupation	What was their occupation at the time of the study?	Supplement III
4. Gender	Was the researcher male or female?	Page 7
5. Experience and training	What experience or training did the researcher have?	Supplement III
Relationship with participants		
6. Relationship established	Was a relationship established prior to study commencement?	Supplement III
7. Participant knowledge of the interviewer	What did the participants know about the researcher? e.g. personal goals, reasons for doing the research	Supplement III
8. Interviewer characteristics	What characteristics were reported about the inter viewer/facilitator? e.g. Bias, assumptions, reasons and interests in the research topic	Supplement III

**Domain 2: study design**		
Theoretical framework		
9. Methodological orientation and Theory	What methodological orientation was stated to underpin the study? e.g. grounded theory, discourse analysis, ethnography, phenomenology, content analysis	Page 7/8
Participant selection		
10. Sampling	How were participants selected? e.g. purposive, convenience, consecutive, snowball	Page 6
11. Method of approach	How were participants approached? e.g. face-to-face, telephone, mail, email	Supplement III
12. Sample size	How many participants were in the study?	Page 8
13. Non-participation	How many people refused to participate or dropped out? Reasons?	Page 8
Setting		
14. Setting of data collection	Where was the data collected? e.g. home, clinic, workplace	Page 7
15. Presence of non-participants	Was anyone else present besides the participants and researchers?	Supplement III
16. Description of sample	What are the important characteristics of the sample? e.g. demographic data, date	Page 8
Data collection		
17. Interview guide	Were questions, prompts, guides provided by the authors? Was it pilot tested?	Page 6/7
18. Repeat interviews	Were repeat interviews carried out? If yes, how many?	Supplement III
19. Audio/visual recording	Did the research use audio or visual recording to collect the data?	Page 7

20. Field notes	Were field notes made during and/or after the inter view or focus group?	Supplement III
21. Duration	What was the duration of the interviews or focus group?	Page 8
22. Data saturation	Was data saturation discussed?	Supplement IV
23. Transcripts returned	Were transcripts returned to participants for comment and/or correction?	Supplement III
**Domain 3: analysis and findings**		
Data analysis		
24. Number of data coders	How many data coders coded the data?	Supplement IV
25. Description of the coding tree	Did authors provide a description of the coding tree?	Supplement V
26. Derivation of themes	Were themes identified in advance or derived from the data?	Page 8
27. Software	What software, if applicable, was used to manage the data?	Page 7
28. Participant checking	Did participants provide feedback on the findings?	Supplement III
Reporting		
29. Quotations presented	Were participant quotations presented to illustrate the themes/findings? Was each quotation identified? e.g. participant number	Yes, Page 7 to 14
30. Data and findings consistent	Was there consistency between the data presented and the findings?	Yes, Page 8 to 21
31. Clarity of major themes	Were major themes clearly presented in the findings?	Yes, Page 8 to 21 and Figures 1 and 2
32. Clarity of minor themes	Is there a description of diverse cases or discussion of minor themes?	Yes, Page 8 to 21

Supplement II: Interview List

Theme 1: Interprofessional collaboration

1. Which type of healthcare professionals were involved in the care process and during the last months?
   A. How did you experience the involvement of these healthcare professionals?
2. How were you involved in the care process?
   A. Did you feel that you had control in the care process?
   B. Did you feel acknowledged as informal caregiver?
   C. Did the healthcare professionals ask for your experiences in the care process?
   D. Did the healthcare professionals consider your advices?
3. Did one person coordinate the care?
   A. How did information transfer occur between healthcare professionals?
   B. Did you often have to repeat information to healthcare professionals?
4. What is your general impression of the collaboration between healthcare professionals?
   A. How was the collaboration between healthcare professionals visible for you?
   B. Did the healthcare professionals know which task they have?
   C. Was the communication process between healthcare professional optimal according to you?
   D. Where the collaboration agreements written down?
   E. Did all healthcare professionals execute the collaboration agreements in the same way?
5. What would you like to change in the collaboration process?
   A. Do you have any advices?

Theme 2: Transmural collaboration

In case relatives with dementia died in a nursing home

1. How did you experience the nursing home transition?
   A. How did you and your relative experience the admission day?
   B. Where you and your relative prepared for the admission? If yes, please explain in which way you were prepared.
   C. Could the admission be prevented according to you?
   D. How were you as loved one involved with the admission?
2. Where the healthcare professionals in the nursing home prepared for the admission of your relative with dementia?
   A. Did you have the impression that they were aware of the healthcare situation of your relative at the admission?
   B. Was the transition process coordinated by one person?
   C. Was it clear for you who your first contact person was?
   D. Could you ask the first contact person everything?
3. How did you experience the handover communication?
   E. Who was responsible for the handover, according to you?
   F. Did you experience the handover as warm, personal and complete?
   G. Are there any factors of which you believe were during this handover?
4. What would you wish to change in the transition process?
   A. Are there any advices that you would like to give to the healthcare professionals?

In case relatives with dementia died at home

1. Were there any preparations made prior to the nursing home admission?
   A. If there were any preparations, could you please explain which kind of preparations you had made and whether you had a conversation with a healthcare professional about this topic?

   B. What was the reason for admission?
2. Where the healthcare professionals aware of your burdensome as loved one?
   A. How did you notice their awareness?
   B. Did you feel overburdened?
   H. How did the healthcare professionals cope with your burdensome?
3. How could the healthcare professionals support you in the entire process?
   A. What would you like to change in the collaboration process?
   B. Do you have any advices?

Supplement III: Additional information about data collection

Nurses from the participating care organizations informed potential candidates about the study through phone calls, or by post through information letters and flyers. The interviewers had no initial contact with the participants during the recruitment procedure, and were only allowed to approach potential candidates when they provided oral or written consent to the nurses.

S.B. (MSc in psychology) and L.V. and S.P. (MSc in nursing) received trainings on the job in performing interviews through piloting and receiving advices from the DEDICATED research team. L.V. was an intern and S.B. and S.P. were PhD students at the DEDICATED project. Prior to asking the interview questions, S.B and S.P. shortly introduced themselves and explained the reasons and aims for the study. They expressed their interest in performing research in the elderly care and for this study, identifying the experiences of informal caregivers. No field notes were made during the interviews. In case S.B. or S.P. could not be present and function as observer, a research assistant or project member of DEDICATED replaced them. None of the interviewers knew the participants. No transcripts were returned to participants for comments or corrections. Apart from the interviewers and informal caregivers, no one else was present. Finally, no repeat interviews were performed.

Supplement IV: Stages in data analysis

The critical realist approach consists of several steps, namely defining the research question that is guided by theory, collecting data, coding data and identifying demi-regularities (key patterns in data), abduction (theoretical redescription of the outcomes) and retroduction (causal mechanisms of the demi-regularities). Prior to the data

analysis, we defined the research question *'How did loved informal caregivers experience IPC with healthcare professionals and how did informal caregivers perceive IPC among healthcare professionals?'*. This division of two collaboration levels was described and explored by Stephan et al. (2015) (Stephan, Möhler, Renom-Guiteras, & Meyer, 2015). We used this study as theoretical guidance to explore IPC with informal caregivers. The coding procedure was executed in two stages. In the first stage, C.K. and I.M. read the first transcript together, identified meaningful segments in the data, assigned primary codes to the segments using the first cycle coding strategies (such as structural coding) of Saldana et al. to optimize unitization, and processed those in the preliminary codebook. In the second stage, all 32 transcripts were coded in four cycles, which resulted in four codebook versions. In each cycle, C.K. and I.M. independently coded eight transcripts using both the first and second cycle coding strategies (such as axial coding) described by Saldana et al. and produced their own coding list, discussed both coding lists and adapted the previous versions of the codebook via negotiated agreements to increase the discriminant capability of the codebook (Saldana, 2013). In the last stage, C.K. and I.M. used the definitive codebook to order the themes, sub-themes and key quotes in Table 1. For the development of the definitive codebook, the template outlined in Macqueen et al. was utilized (Macqueen, McLellan-Lemal, Kay, Milstein, & Cdc, 1998). During all coding cycles, researchers C.K. and I.M. redefined and re-described our categories, codes, sub-codes based on existing theories and concepts in literature (abduction), and consultation with DEDICATED research team to improve the investigator triangulation process, and thereby the validity of the analysis. The identified demi-regularities were types of involvement, insight into structural collaboration, and perceived collaboration outcomes. An example of abduction concerned the deciding what constitutes shared decision-making as previous literature showed that decision-making occurs for minor as well as major decisions, which also includes nursing home transitions. At the end we carried out retroduction, in which we identified factors influencing the types of involvement and the information exchange among healthcare professionals. Afterwards, codes and sub-codes have been rephrased

three times (together with DEDICATED research team). We also identified quotations that underline the outcomes, represent how both the majority and minority of the informal caregivers expressed themselves, and increase the readability of the results (Eldh, Årestedt, & Berterö, 2020). These key quotes are shown in the results. Both code saturation (identifying of new codes) and meaning saturation (identifying new meanings of the developed codes) was reached during the third coding cycle (Hennink, Kaiser, & Marconi, 2017). Participants were not asked to provide feedback on the results.

References

Eldh, A. C., Årestedt, L., & Berterö, C. (2020). Quotations in Qualitative Studies: Reflections on Constituents, Custom, and Purpose. *International Journal of Qualitative Methods, 19*, 1609406920969268. doi:10.1177/1609406920969268

Hennink, M. M., Kaiser, B. N., & Marconi, V. C. (2017). Code Saturation Versus Meaning Saturation:How Many Interviews Are Enough? *Qualitative Health Research, 27*(4), 591-608. doi:10.1177/1049732316665344

Macqueen, K., McLellan-Lemal, E., Kay, K., Milstein, B., & Cdc, A. (1998). *Codebook Development for Team-Based Qualitative Analysis* (Vol. 10).

Saldana, J. (2013). *The Coding Manual for Qualitative Researchers*. Thousand Oaks, California SAGE.

Stephan, A., Möhler, R., Renom-Guiteras, A., & Meyer, G. (2015). Successful collaboration in dementia care from the perspectives of healthcare professionals and informal carers in Germany: results from a focus group study. *BMC Health Serv Res, 15*(1), 208. doi:10.1186/s12913-015-0875-3

Themes	***Categories***	***Codes***
**1. Informal caregivers' roles in IPC with healthcare professionals**	1A. Types of informal caregivers' roles	1A. I Information exchai
		1A. II Care process
		1A. III Shared decision-making

	1B. Factors influencing informal caregivers' roles	1B. I Intrinsic factors
		1B. II Extrinsic factors
**2. Informal caregivers' perception of IPC among healthcare professionals**	2A. Visible collaboration process	2A. I Communication pr
		2A. II Team process
	2B. Effecting collaboration outcomes	2B. I Information excha
		2B. II Care process
	2C. Factors influencing collaboration outcomes	2C. Micro-level factors
		2C. Macro-level factors
